# Variations in Community Prevalence and Determinants of Recreational and Utilitarian Walking in Older Age

**DOI:** 10.1155/2015/382703

**Published:** 2015-08-03

**Authors:** Elizabeth Procter-Gray, Suzanne G. Leveille, Marian T. Hannan, Jie Cheng, Kevin Kane, Wenjun Li

**Affiliations:** ^1^Health Statistics and Geography Lab, Division of Preventive and Behavioral Medicine, University of Massachusetts Medical School, 55 Lake Avenue North, Worcester, MA 01655, USA; ^2^College of Nursing and Health Sciences, University of Massachusetts Boston, 100 Morrissey Boulevard, Boston, MA 02125-3393, USA; ^3^Institute for Aging Research, Hebrew SeniorLife/Harvard Medical School and Beth Israel Deaconess Medical Center, 1200 Centre Street, Boston, MA 02131-1097, USA

## Abstract

*Background*. Regular walking is critical to maintaining health in older age. We examined influences of individual and community factors on walking habits in older adults. *Methods*. We analyzed walking habits among participants of a prospective cohort study of 745 community-dwelling men and women, mainly aged 70 years or older. We estimated community variations in utilitarian and recreational walking, and examined whether the variations were attributable to community differences in individual and environmental factors. *Results*. Prevalence of recreational walking was relatively uniform while prevalence of utilitarian walking varied across the 16 communities in the study area. Both types of walking were associated with individual health and physical abilities. However, utilitarian walking was also strongly associated with several measures of neighborhood socioeconomic status and access to amenities while recreational walking was not. *Conclusions*. Utilitarian walking is strongly influenced by neighborhood environment, but intrinsic factors may be more important for recreational walking. Communities with the highest overall walking prevalence were those with the most utilitarian walkers. Public health promotion of regular walking should take this into account.

## 1. Introduction

The benefits of regular physical activity for older adults, including reduced risk of chronic diseases and disabilities, are well known [1–6]. The Centers for Disease Control and Prevention (CDC) currently recommends 150 minutes per week of moderate-intensity (e.g., brisk walking) or 75 minutes of vigorous-intensity aerobic activity per week for adults aged 65 and older [7]. However, the most recent CDC data indicate that only 15.9% of American older adults met these guidelines in 2011 [7] and are far from reaching physical activity goals set by Healthy People 2020 [8].

Walking is the most common type of moderate-intensity physical activity among older adults [9–11]. It is inexpensive and achievable by people of a wide range of physical abilities. Walking may be done for two primary purposes: (1) for intentional exercise and recreation or (2) simply as a means of getting from place to place for utilitarian purposes (e.g., to accomplish errands or get to work). The biomechanics, optimal walking speed, and energy expenditure of these two types of walking may differ, as load or gradient differs [12–14]. Several previous studies have investigated how recreational walking and utilitarian walking are influenced by the walking environment [15–17]. Li et al. found that utilitarian walkers had significantly higher risks of falling and being injured in falls than recreational walkers [18]. However, to our knowledge, no study has looked at the wider range of determinants specifically to see how they might differ between the two types of walking, such as walkers' physical abilities, socioeconomic factors, environmental features, and neighborhood characteristics.

We examined the recreational and utilitarian walking habits of older adults living in 16 neighborhoods in the greater Boston, Massachusetts area. We investigated the extent and determinants of community-level variations in walking frequencies, and possible implications to public health practices.

## 2. Materials and Methods

### 2.1. Study Design and Participants

The MOBILIZE Boston Study has been described in detail elsewhere [18–20]. Briefly, it is a prospective cohort study to investigate risk factors and mechanisms of falls among community-dwelling persons, mainly aged 70 years and older. The cohort included 765 participants living in the Boston, MA area, who are able to read and speak in English and walk 20 feet without the assistance of another person, intending to stay in the Boston area for at least two years, and who are without moderate to severe cognitive impairment (score ≥18 on the Mini-Mental State Exam (MMSE)) [21]. Participants were recruited from September 2005 to December 2007, using door-to-door recruitment in randomly sampled households within 5 miles of the study clinic at Hebrew SeniorLife in Boston with at least one member aged 70 years or older as recorded in city and town lists [19]. Domestic companions aged 64 and older living with study participants (aged ≥ 70 years) were eligible to enroll in the study. This analysis excluded 20 participants who did not have data on walking habits or on covariates used in the analyses, for a final sample size of 745 participants. Written informed consent was obtained from study participants. The Institutional Review Board of Hebrew Senior Life approved this study.

### 2.2. Data Collection

The two-part baseline assessment included a home interview, followed within 4 weeks by a study clinic visit. During the baseline home interview, trained interviewers administered questionnaires to assess self-rated general health, cognitive function [21], physical function [22], health behaviors [23], fall history in the 12 months prior to baseline, medication use, comorbid conditions [24, 25], and sociodemographic characteristics.

During the home visit, participants were given a questionnaire to complete and bring to their clinic visit. The form was reviewed by research assistants for completion. On the questionnaire, participants were asked about their walking habits. The frequency of outdoor walking was assessed with the question, “Over the past seven days, how often did you take a walk outside your home or yard for any reason (never, seldom (1-2 days), sometimes (3-4 days), or often (5–7 days))?” Utilitarian walking was determined by the query, “Do you walk to the store, post office, bank or other businesses in your neighborhood?” Recreational walking was queried as, “Besides walking to stores or businesses, do you walk for exercise in your neighborhood?” The frequencies of utilitarian and recreational walking were categorized as “Never, less than once per month, 1–3 times per month, 1-2 times per week, and 3 or more times per week.” For the purpose of the current analysis, three operational outcomes of walking habits were defined, namely, “habitual” walking (walking outside the home for any purpose five or more days per week), recreational walking at least once per week, and utilitarian walking at least once per week.

During the clinical examination at baseline, participant body height and weight were assessed to calculate body mass index (BMI, kg/m^2^). Balance was measured with the Berg Balance Test [26]. An inability to stand from a chair without using arms was taken as an indicator of poor lower extremity muscle strength. Lower extremity function was assessed by the Short Physical Performance Battery (SPPB) [27, 28]. Gait speed (m/sec) was assessed by the shortest time taken to complete 2 trials of a 4-meter walk at self-selected walking speed.

### 2.3. Characteristics of Communities

**Figure 1:**
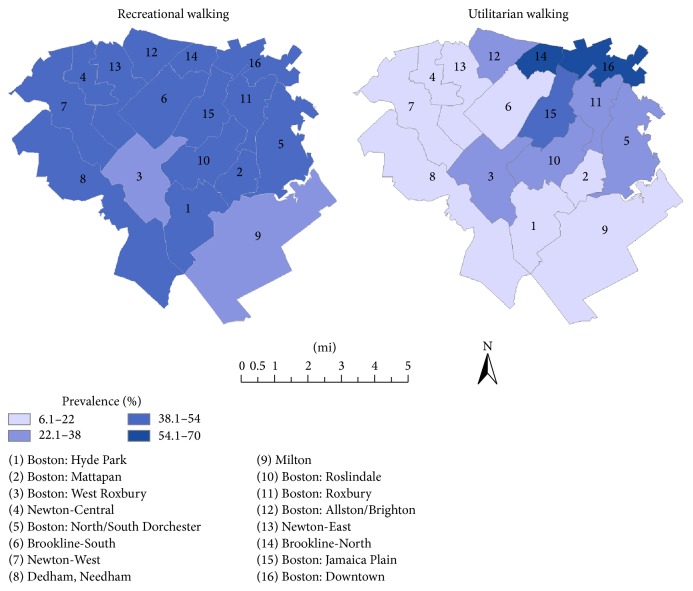
Community variations in prevalence of recreational and utilitarian walking.

Participants lived in Boston and a number of neighboring towns. A participant's community was defined as the town, city, or subdivision of a large city of residence. The city of Boston includes 16 communities according to neighborhood planning districts defined by the Boston Redevelopment Authority. In order to ensure the compatibility of community sizes and adequate sample sizes for each geographic unit of analysis, we combined several adjacent communities with similar sociodemographic profiles into a single community unit. Larger cities with larger sample sizes were divided into smaller units based on established geographic concepts (frequently used neighborhood names). As shown in Figure 1, 16 communities were defined for the purpose of this analysis.

Each of the participants' home addresses was geocoded using ArcGIS Desktop 10 (ESRI 2011, Redlands, CA) and their census blocks were determined by linking the geographic coordinates to US Census block maps. Participants' census block groups and census tracts were identified accordingly. Community-level demographic and SES data were derived by aggregating census track level data from 5 year rolling averages of 2005–2009 American Community Surveys of US Census. Participants' community-level characteristics of interest included measures of income, educational attainment, housing characteristics, community stability, and racial composition.

Measures of geographic access to community resources were derived using Geographic Information System (GIS) based road network distances to community amenities. From the Massachusetts Geographic Information System (MassGIS), we obtained GIS data layers of libraries (2005), town halls (2013), public parks (2014), transportation infrastructure (2012), and land use and housing density data (2010). GIS layer of food stores was obtained from InfoUSA (2012) and GIS layer of post offices, from United States Post Office (2014). Using ArcGIS 10.1 (ESRI, Redlands, CA), we calculated the road network distance in miles from the participant's residence to the closest amenity of each type and residential density as number of households per acre of residential land.

### 2.4. Statistical Analysis

Participant characteristics found to be associated with at least one of the walking outcomes of interest in previous studies [18] were summarized with descriptive statistics. Their associations with the three walking outcomes were estimated using unadjusted logistic regressions.

Multivariable logistic regression models adjusted for the personal composite score were used to estimate associations of selected community socioeconomic factors and amenity distances with the three walking outcomes. These measures were treated as linear continuous variables except for percentage of vacant housing units and percentage minority. Because of their U-shaped associations with utilitarian walking, we constructed three categories of each of these variables. We modeled participants in the same community as a cluster for the socioeconomic measures, because they shared the same SES values, but each participant had his/her own unique amenity-distance scores. A seemingly-unrelated-estimation test [29, 30] was used to assess the equality of the associations (odds ratios) of these community characteristics and distance-to-amenities measures with recreational versus utilitarian walking.

Community level differences in prevalence of walking habits were assessed using logistic regression models, where each of the three walking habit variables was the dependent variable, and community indicators were predictors. Four models were estimated for each walking habit: (1) community differences without adjustment; (2) with adjustment for personal characteristics; (3) with adjustment for personal characteristics and distances to amenities; and (4) with adjustment for personal characteristics, amenity distances, and community-level SES factors. In order to preserve degrees of freedom but still adjust for these factors, we derived a single composite adjustment score for each walking outcome for each individual using the entire set of characteristics as predictors in logistic models [22, 31]. A person's composite score was calculated as the summation of all the products of each specific regression coefficient multiplied by his/her value of the corresponding predictor. The area under the ROC (Receiver Operator Characteristic) curves of each model was used as a measure of model fit. A comparison between those participants aged 64 to 79 years and those 80 years and older revealed very similar community-level trends, so all ages were combined in the analysis. All statistical analyses were carried out using Stata MP 12 (Stata Corp LP, College Station, TX).

## 3. Results

One in three participants reported walking at least five days per week outside the home; 42% walked recreationally at least once a week; and 28% walked for utilitarian purposes at least once a week. About 18.3% walked for both utilitarian and recreational purposes at least once a week. Mean self-selected walking speed for this population was 0.92 m/sec for women and 0.99 m/sec for men.

The associations of selected personal characteristics with the three walking outcomes under investigation were strong, especially so with habitual walking (Table 1). Associations with the specific behaviors of recreational and utilitarian walking were less robust but were similar to each other, especially for traits related to physical impairments such as slow walking speed (<0.68 m/sec), poor lower-body strength, and poor balance which may make walking difficult or more dangerous.

Despite the fact that fairly similar determinants at the personal level. Recreational and utilitarian walking showed very different patterns of association with community-related factors, the communities differed greatly in the average walking distances to the nearest bus stop, subway, hospital, shopping center, post office, park, food store, town hall, and library, and each of these distances was negatively associated with utilitarian walking (*p* < 0.01, Table 2). None was associated with recreational walking, however. The difference between utilitarian walking and recreational walking with respect to these destination distances was significant in every case. Walking at least five days per week was associated with shorter distances to the nearest subway stop, hospital, post office, and food store (Table 2).

As with amenity distances, community SES characteristics were more likely to be associated with utilitarian walking than with recreational walking. Utilitarian walking was significantly associated with a lower community median household income, a lower proportion of owner-occupied housing units, and intermediate percentages of vacant housing units (5–10%) and minority population (20–50%) (Table 2). With the exception of median income, each of the above factors associated with utilitarian walking was thereby also associated with the likelihood of walking five or more days per week, but none of the factors was significantly associated with recreational walking.

A comparison of the prevalence of habitual walking across the 16 Boston-area communities showed great variation, ranging from 16.7% in Hyde Park to 48.1% in Boston Downtown (Table 3). This variation appeared to result mainly from community differences in utilitarian rather than recreational walking (Figure 1). Both before and after adjustment for personal characteristics (Table 3, Models 1 and 2), there were significant differences among communities in utilitarian walking at least once per week (*p* < 0.001 overall) but not in recreational walking (*p* > 0.85).

Further adjustment for distance from home to amenities diminished the magnitudes of community associations with utilitarian walking somewhat, such that the community variable was no longer a significant predictor overall (*p* = 0.10, Table 3, Model 3), and the final adjustment for community SES greatly decreased the overall association (*p* = 0.94, Table 3, Model 4), indicating that access to amenities and socioeconomic factors had accounted for a great deal of the community variation in utilitarian walking. Even after all adjustments, however, the odds ratios for community differences from the referent community remained higher in magnitude for utilitarian walking (range: 1.90–6.96) than for recreational walking (range: 0.72–1.14). Measures of community associations with walking at least five days per week (habitual) were diminished by adjustment for distance to amenities and community SES in a way similar to that of utilitarian walking, showing the importance of utilitarian walking in producing the patterns of total walking observed but community differences remained significant overall (*p* = 0.01, Table 2, Model 4). The three communities with the highest percentages of elders who walked five or more days per week for any purpose (Brookline North, Jamaica Plain, and Boston Downtown) also had the highest percentages of persons who walked at least once per week for utilitarian purposes, and they were the only communities with more utilitarian than recreational walkers.

Areas under the ROC curve, indicating the goodness-of-fit of models in Table 3, show that community alone (Model 1) was a stronger predictor of a person's utilitarian walking habit (area = 0.70) than of recreational walking habit (area = 0.56, *p* < 0.001). Adjustments for personal characteristics and distances improved each model significantly, but the fit of the recreational walking model never became as good as that for utilitarian walking (*p* < 0.001 for each model).

## 4. Discussion

This study found variability among communities in the percentage of elders walking five or more times per week. Much of this variability could be attributed to differences in utilitarian rather than recreational walking habits. Prevalence of recreational walking at least once per week was low (40%) among elders in the area, regardless of socioeconomic and built environmental conditions. Prevalence of utilitarian walking was also generally low (28% overall) but varied significantly among communities, accounting for much of the difference in the percentage of elders walking regularly. The data imply, on one hand, the importance of utilitarian walking, and on the other hand, the limited contribution of recreational walking, to total physical activity.

While many personal characteristics, especially those concerning physical abilities and disabilities, were associated with recreational (and utilitarian) walking, we did not find any socioeconomic or environmental factors that were significant predictors of recreational walking. In contrast, utilitarian walking was strongly associated with many community characteristics including shorter walking distances from home to several amenities and neighborhood socioeconomic factors such as lower income and lower housing occupancy. This agrees with the findings for general adult populations [15, 32] and for older adults [16]. Van Holle and associates [16] made objective measures of the walkability of neighborhoods and found that neighborhood characteristics were associated with “walking for transport” or “walking for errands” but not with walking for recreation. It is worth noting that we found the same patterns of association and nonassociation of community characteristics with walking of the three types among those participants aged 80 and older as well as among those aged 64 to 79 years.

This analysis has several strengths. First, this study examined walking habits in a large cohort of community-dwelling elders aged 70 and above, with 37% aged 80 and older, a segment of the population that has been little studied with regard to walking. This older age of our cohort may account for the somewhat slower mean gait speed than that reported in the literature for healthy septuagenarians [33]. The MOBILIZE Boston participants were strategically recruited to represent a broad range of socioeconomic and residential conditions; the cohort resembled the underlying population well [19]. We obtained detailed measurement of a wide range of personal and socioeconomic characteristics and distances to amenities that might attract walkers in a well-characterized urban area. The distinction between walking for recreational versus utilitarian purposes afforded the opportunity to identify two different sets of determinants specific to the two types of walking, filling a knowledge gap in the elder population.

This study also has several limitations. It is limited to one urban area in the northeastern region of the country where the older adult population tended to be English-speaking, of white race and have higher income. Although the MOBILIZE Boston cohort matched the general older population according to sex, race, and ethnicity, the participants had somewhat higher education than the general community. Self-report of walking habits was subject to recall inaccuracies. Still, relative frequencies across communities should be fairly accurate, especially after adjustment for a wide range of demographic characteristics. Duration of walking activity was not collected except in very broad categories which did not allow for community-level or person-level comparisons. We also did not know whether or how often a load might have been carried by walkers. One might infer that this would be more common among utilitarian walkers (while shopping, etc.), possibly adding a physiological benefit of extra energy expenditure [13] but increasing risk of injury in a fall [34].

The unmeasured potential neighborhood self-selection and the cross-sectional design do not allow examination of longitudinal changes in walking habits which might elucidate causality. In addition, information on selection of walking routes was not collected. To address these issues, our current studies have been collecting detailed information on time and location of walking and selection of walking paths among older adults, using accelerometer and Global Positioning System devices.

Clearly, an older person's decision to walk regularly depends on a whole host of factors in addition to the built environment: necessary errands if they do not drive, access to public transportation, physical ability, time availability, feelings of fear, culture, and social influences being but a few [35]. However, the benefits of physical activity, in preventing obesity, arthritis, and their associated health problems and especially in warding off disability as people age [1–5], suggest that efforts to encourage walking to the extent possible by modifications of the environment may be worthwhile. Our findings point toward further investigation of communities like Brookline North, Jamaica Plain, and Downtown Boston to see which features distinguish them from other Boston communities in promoting physical activity by elders.

## 5. Conclusions

The primary finding of this study was that while the prevalence of recreational walking was relatively uniform across all communities, the prevalence of utilitarian walking varied widely and was more strongly associated with multiple factors at both the community and individual levels. In most communities examined in this study, the prevalence of regular walking behavior was low, which suggests a tremendous opportunity for health promotion. The challenge for public health is not only to find ways to promote utilitarian walking by elders using the knowledge gained to date but also to increase our understanding of factors that encourage recreational walking, so that both types of walking become more appealing, accessible, and safe.

## Figures and Tables

**Table 1:** Participants' characteristics by walking habits.

	Mean (SD) or %						
	Overall (*n* = 745)	Walking ≥5 days/week		Recreational walking ≥once/week		Utilitarian walking ≥once/week	
		Yes (*n* = 251)	No (*n* = 494)	Yes (*n* = 310)	No (*n* = 429)	Yes (*n* = 208)	No (*n* = 530)
Demographic							
Age (years)	78.1 (5.4)	77.2 (5.2)^*∗∗*^	78.6 (5.5)	77.4 (5.2)^*∗∗*^	78.6 (5.5)	76.9 (5.0)^*∗∗*^	78.5 (5.5)
Male gender	36.0	43.3^*∗∗*^	32.2	37.1	35.0	38.5	34.9
White race/ethnicity	77.9	80.5	76.5	81.3	77.4	78.4	77.7
Education							
High school graduate or less	34.1	28.7^*∗*^	36.8	31.3	36.4	33.7	34.5
Some college or college graduate	34.5	34.7^*∗*^	34.4	34.2	34.3	32.7	34.9
Graduate studies	31.4	36.7^*∗*^	28.7	34.5	29.4	33.7	30.6
Lifestyle							
Body mass index (kg/m^2^)							
<25	32.1	37.9^*∗∗*^	29.2	35.2^*∗∗*^	29.8	37.5^*∗*^	30.0
25–29.9	41.7	44.2^*∗∗*^	40.5	44.5^*∗∗*^	40.1	43.3^*∗*^	41.5
30+	26.2	17.9^*∗∗*^	30.4	20.3^*∗∗*^	30.1	19.2^*∗*^	28.5
Use of alcohol							
Every day use	13.3	19.5^*∗∗*^	10.1	12.9	13.5	16.8	12.1
1–6 days/week	25.4	29.1^*∗∗*^	23.5	29.7	22.4	26.4	24.9
1–3 days/month	22.0	24.7^*∗∗*^	20.7	20.7	23.1	20.7	22.6
Not at all	39.3	26.7^*∗∗*^	45.8	36.8	41.0	36.1	40.4
Physical disability							
Balance (Berg score)							
<48	21.6	13.2^*∗∗*^	25.9	13.9^*∗∗*^	27.3	15.9^*∗∗*^	23.6
48–50	25.1	23.1^*∗∗*^	26.1	26.1^*∗∗*^	24.0	19.2^*∗∗*^	27.2
51+	53.3	63.8^*∗∗*^	48.0	60.0^*∗∗*^	48.7	64.9^*∗∗*^	49.3
Unable to do chair-stand test without arms	7.5	4.4^*∗*^	9.1	2.9^*∗∗*^	10.7	2.4^*∗∗*^	9.4
Gait speed (m/sec)							
<0.68	13.3	6.0^*∗∗*^	17.0	8.7^*∗∗*^	16.6	3.9^*∗∗*^	16.8
0.68–1.33	80.1	82.5^*∗∗*^	79.0	81.3^*∗∗*^	79.3	87.0^*∗∗*^	77.6
>1.33	6.6	11.6^*∗∗*^	4.0	10.0^*∗∗*^	4.2	9.1^*∗∗*^	5.7
Activities of daily living							
No difficulty	77.9	84.1^*∗*^	74.7	83.9^*∗∗*^	73.7	85.6^*∗∗*^	75.3
Little/some difficulty	14.9	11.2^*∗*^	16.8	10.0^*∗∗*^	18.2	11.1^*∗∗*^	16.0
Much difficulty/inability	7.3	4.8^*∗*^	8.5	6.1^*∗∗*^	8.2	3.4^*∗∗*^	8.7
Short physical performance battery <10	40.5	30.3^*∗∗*^	45.8	32.3^*∗∗*^	45.9	27.4^*∗∗*^	45.1
Reduced activity due to illness past year	28.3	25.9	29.6	29.4	27.5	23.6	30.0
Other health-related							
Moderate/severe bodily pain	39.1	29.9^*∗∗*^	43.7	34.5^*∗*^	42.4	35.6	40.4
Number of comorbid conditions	2.9 (1.5)	2.7 (1.4)^*∗*^	3.0 (1.5)^*∗*^	2.8 (1.5)	2.9 (1.5)	2.6 (1.4)^*∗∗*^	3.0 (1.5)
Fair/poor self-rated health	14.4	7.2^*∗∗*^	18.0	11.6	15.6	10.6	15.5
Peripheral neuropathy	12.1	7.2^*∗∗*^	14.6	9.4	14.0	7.7^*∗*^	13.6
Foot pain	23.9	18.7^*∗*^	26.5	22.3	25.2	23.1	24.2
Number of medications							
0–4	34.9	41.4^*∗∗*^	31.6	38.1	32.9	45.2^*∗∗*^	31.1
5–8	45.2	43.8^*∗∗*^	46.0	43.6	46.9	39.9^*∗∗*^	47.4
9+	19.9	14.7^*∗∗*^	22.5	18.4	20.3	14.9^*∗∗*^	21.5
Impaired cognition (MMSE 18–23)	11.7	7.2^*∗∗*^	14.0	10.3	12.4	9.1	12.3
Falls efficacy score <90	13.6	7.6^*∗∗*^	16.6	12.9	13.8	11.1	14.3

^*∗*^ *p* < 0.05 in unadjusted logistic regression of the walking behavior on the characteristic. In the case of multicategory characteristics, for example, education level, the statistic applies to the characteristic overall, not to particular categories.

^*∗∗*^ *p* < 0.01.

**Table 2:** Associations of three measures of walking with geographic access to amenities and community-level socioeconomic characteristics.

Characteristic	Community mean (SD) or percent	Community range (min., max)	Odds ratio (95% confidence interval)^1^			SUEST test^2^ *p*-value
			Walk at least 5 days per week	Recreation walk at least once per week	Utilitarian walk at least once per week	
Mean distances (km) from home block centroid to nearest amenities						
Bus stop	0.48 (0.31)	0.20, 1.40	0.74 (0.52–1.04)	1.04 (0.76–1.43)	0.25 (0.15–0.43)^*∗∗*^	<0.001
Subway	2.03 (1.42)	0.47, 5.23	0.88 (0.79–0.97)^*∗∗*^	0.93 (0.85–1.02)	0.69 (0.61–0.78)^*∗∗*^	<0.001
Hospital	2.14 (1.41)	0.56, 4.80	0.84 (0.76–0.94)^*∗∗*^	0.92 (0.84–1.02)	0.69 (0.60–0.78)^*∗∗*^	<0.001
Shopping center or mall	4.36 (1.71)	1.16, 7.32	0.93 (0.85–1.02)	1.00 (0.92–1.08)	0.85 (0.77–0.94)^*∗∗*^	0.002
Post office	1.89 (1.17)	0.73, 4.75	0.82 (0.73–0.92)^*∗∗*^	0.92 (0.83–1.02)	0.64 (0.55–0.74)^*∗∗*^	<0.001
Public park (≥1 acre)	0.57 (0.23)	0.28, 1.10	0.77 (0.53–1.13)	0.82 (0.58–1.16)	0.41 (0.26–0.64)^*∗∗*^	0.02
Grocery/convenience store	0.78 (0.44)	0.22, 1.65	0.77 (0.61–0.99)^*∗*^	1.08 (0.87–1.34)	0.26 (0.17–0.39)^*∗∗*^	<0.001
Town hall	3.69 (1.44)	1.49, 7.09	0.93 (0.86–1.02)	0.97 (0.89–1.05)	0.85 (0.77–0.94)^*∗∗*^	0.03
Public library	1.18 (0.54)	0.62, 2.49	0.95 (0.76–1.18)	1.02 (0.83–1.26)	0.65 (0.50–0.83)^*∗∗*^	0.002

Community-level socioeconomic factors						
Median household income ($10,000)	7.9 (3.3)	2.9, 12.7	0.95 (0.90–1.01)	0.98 (0.94–1.03)	0.87 (0.77–0.99)^*∗*^	<0.001
% below federal poverty level	9.3 (8.1)	1.5, 28.8	1.25 (0.99–1.57)	1.07 (0.88–1.30)	1.66 (1.00–2.77)	0.007
% of adults unemployed	19.0 (5.1)	11.8, 28.4	1.07 (0.71–1.61)	1.12 (0.81–1.53)	1.06 (0.43–2.62)	0.38
% college graduates	54.4 (24.2)	12.3, 82.0	1.01 (0.92–1.10)	1.00 (0.94–1.07)	1.01 (0.83–1.22)	0.88
% of housing units owner-occupied	56.3 (21.5)	23.7, 89.2	0.89 (0.82–0.97)^*∗∗*^	0.96 (0.89–1.03)	0.73 (0.63–0.86)^*∗∗*^	<0.001
% housing units vacant, referent <5%	6.5 (2.9)	3.1, 12.5	Ref.	Ref.	Ref.	
5–10%			1.59 (1.11–2.29)^*∗*^	1.29 (0.92–1.82)	3.14 (1.42–6.97)^*∗∗*^	<0.001
>10%			1.31 (0.83–2.07)	1.21 (0.79–1.84)	2.05 (0.75–5.61)	
% minority (non-White), referent <20%	34.9 (28.0)	9.6, 93.9	Ref.	Ref.	Ref.	
20–50%			1.63 (1.13–2.36)^*∗∗*^	1.36 (0.96–1.93)	3.98 (1.85–8.54)^*∗∗*^	<0.001
>50%			1.06 (0.69–1.63)	1.25 (0.84–1.85)	1.74 (0.73–4.13)	

^*∗*^ *p* < 0.05.

^*∗∗*^ *p* < 0.01.

^1^Odds ratio estimates adjusted for personal characteristics.

^2^Seemingly-unrelated estimation testing the hypothesis that the odds ratio in the recreational walking regression equals that in the utilitarian walking regression.

**Table 3:** Personal walking habits of participants by community.

	*N*	Prev. (%)	Model 1 unadjusted		Model 2 adjusted for personal characteristics^2^		Model 3 adjusted for personal and distance to amenities^3^		Model 4 adjusted for personal, distance, and community SES^4^	
			OR^1^	95% CI	OR	95% CI	OR	95% CI	OR	95% CI
(A) Walking ≥5 days/week										
Boston: Hyde Park	36	16.7	Ref.		Ref.		Ref.		Ref.	
Boston: Mattapan	24	25.0	1.67	0.47–5.96	2.02	0.54–7.62	2.82	0.74–10.79	2.36	0.62–8.93
Boston: West Roxbury	71	25.4	1.70	0.61–4.74	1.96	0.67–5.68	2.57	0.87–7.57	2.40	0.81–7.17
Newton-Central	27	25.9	1.75	0.51–5.98	1.92	0.52–7.06	2.45	0.66–9.06	1.88	0.50–6.99
Boston: North/South Dorchester	60	26.7	1.82	0.64–5.18	2.39	0.80–7.12	2.25	0.74–6.80	2.08	0.69–6.28
Brookline-South	51	31.4	2.29	0.79–6.58	1.70	0.57–5.10	1.41	0.46–4.26	1.03	0.33–3.26
Newton-West	73	31.5	2.30	0.84–6.29	1.87	0.66–5.29	3.29	1.15–9.45^*∗*^	2.48	0.85–7.23
Dedham, Needham	40	32.5	2.41	0.80–7.22	1.95	0.62–6.08	2.90	0.92–9.16	1.63	0.52–5.16
Milton	47	34.0	2.58	0.89–7.48	2.68	0.89–8.11	3.50	1.14–10.76^*∗*^	2.72	0.89–8.31
Boston: Roslindale	37	35.1	2.71	0.90–8.19	3.33	1.04–10.66^*∗*^	3.55	1.09–11.56^*∗*^	3.41	1.05–11.10^*∗*^
Boston: Roxbury	59	35.6	2.76	0.99–7.71	3.31	1.13–9.69^*∗*^	2.82	0.96–8.31	2.23	0.74–6.72
Boston: Allston/Brighton	38	36.8	2.92	0.97–8.73	2.75	0.88–8.56	1.74	0.55–5.49	1.44	0.43–4.78
Newton-East	47	38.3	3.10	1.08–8.92^*∗*^	2.27	0.76–6.82	2.82	0.93–8.54	1.82	0.60–5.57
Brookline-North	51	47.1	4.44	1.58–12.51^*∗∗*^	3.89	1.32–11.50^*∗*^	2.29	0.76–6.85	1.64	0.49–5.44
Boston: Jamaica Plain	57	47.4	4.50	1.62–12.47^*∗∗*^	4.95	1.70–14.43^*∗∗*^	3.37	1.15–9.89^*∗*^	2.66	0.86–8.25
Boston: Downtown	27	48.1	4.64	1.46–14.76^*∗∗*^	6.86	2.01–23.38^*∗∗*^	3.24	0.95–11.09	2.22	0.55–8.99
	745									
Overall *p* value for community differences				*p* = 0.10		*p* = 0.07		*p* = 0.56		*p* = 1.0
Area under ROC curve				0.60		0.73		0.74		0.75
*p* value for change in area under ROC compared to previous model				NA		*p* < 0.001		*p* = 0.004		*p* = 0.64

(B) Walking for recreation ≥1 time/week										
Boston: Hyde Park	36	41.7	1.46	0.64–3.35	1.52	0.65–3.58	1.10	0.46–2.63	0.95	0.39–2.27
Boston: Mattapan	24	41.7	1.46	0.56–3.78	1.70	0.62–4.66	1.36	0.49–3.76	1.14	0.41–3.16
Boston: West Roxbury	70	32.9	Ref.		Ref.		Ref.		Ref.	
Newton-Central	26	38.5	1.28	0.50–3.25	1.14	0.43–3.00	1.04	0.39–2.74	0.79	0.30–2.11
Boston: North/South Dorchester	58	41.4	1.44	0.70–2.97	1.67	0.79–3.54	1.16	0.55–2.48	0.97	0.46–2.08
Brookline-South	50	46.0	1.74	0.82–3.67	1.44	0.66–3.12	0.88	0.40–1.95	0.86	0.39–1.92
Newton-West	73	38.4	1.27	0.64–2.53	1.20	0.59–2.45	1.14	0.55–2.33	1.06	0.52–2.18
Dedham, Needham	40	40.0	1.36	0.61–3.05	1.41	0.60–3.27	1.23	0.53–2.87	1.02	0.44–2.40
Milton	47	34.0	1.05	0.48–2.31	1.01	0.45–2.26	0.78	0.34–1.77	0.72	0.32–1.65
Boston: Roslindale	37	43.2	1.56	0.69–3.53	1.68	0.72–3.92	1.25	0.53–2.94	1.04	0.44–2.46
Boston: Roxbury	59	39.0	1.31	0.63–2.69	1.30	0.62–2.75	0.97	0.46–2.07	0.86	0.40–1.84
Boston: Allston/Brighton	38	47.4	1.84	0.82–4.13	1.59	0.68–3.69	0.96	0.41–2.28	0.99	0.42–2.33
Newton-East	47	53.2	2.32	1.09–4.96^*∗*^	1.72	0.79–3.77	1.33	0.59–2.96	1.00	0.44–2.27
Brookline-North	51	49.0	1.96	0.94–4.13	1.87	0.86–4.05	1.09	0.49–2.42	0.96	0.43–2.14
Boston: Jamaica Plain	56	44.6	1.65	0.80–3.40	1.63	0.77–3.46	0.99	0.46–2.14	0.93	0.43–2.01
Boston: Downtown	27	48.1	1.90	0.77–4.69	1.83	0.71–4.72	1.01	0.39–2.66	0.92	0.35–2.43
	739									
Overall *p* value for community differences				*p* = 0.86		*p* = 0.97		*p* = 1.0		*p* = 1.0
Area under ROC curve				0.56		0.66		0.68		0.68
*p* value for change in area under ROC compared to previous model				NA		*p* < 0.001		*p* = 0.007		*p* = 0.92

^*∗*^ *p* < 0.05.

^*∗∗*^ *p* < 0.01.

^1^Odds ratio estimates for the specified walking habit relative to a member of the referent community, obtained by logistic regression.

^2^Logistic regression model adjusted for a composite score predicted from the following personal characteristics: age, sex, self-rated health, bodily pain, alcohol consumption, education level, body mass index, short physical performance battery, falls efficacy, ADL (activities of daily life ability), race, foot pain, balance, number of comorbidities, gait speed, peripheral neuropathy, flights of stairs in the home, Mini-Mental State Examination Score, number of medications, illness causing reduced activity in past year, and strength to rise from a chair.

^3^Logistic regression model adjusted for a composite score predicted from the personal characteristics as listed in model 2 and for distances from the participant's block centroid to the following amenities: the nearest bus stop, subway station, hospital, shopping center, post office, public park, food store, town hall, and library.

^4^Logistic regression model adjusted for a composite score predicted from the personal and distances-to-amenities characteristics as listed in model 3 and for the following socioeconomic characteristics of the participant's community: median household income, percent below federal poverty level, percent of adults unemployed, percent of college graduates, percent of housing units owner-occupied, percent of housing units vacant, and percent of minority (non-White).

## List of Abbreviations

CDC: the Centers for Disease Control and Prevention, USA
MMSE: the Mini-Mental State Exam
BMI: Body mass index (BMI, kg/m^2^)
SPPB: Short physical performance battery
SES: Socioeconomic status
GIS: Geographic information system
ROC: Receiver operator characteristic.
